# Assessing the suitability of copper thiocyanate as a hole-transport layer in inverted CsSnI_3_ perovskite photovoltaics

**DOI:** 10.1038/s41598-018-33987-7

**Published:** 2018-10-24

**Authors:** Anjana Wijesekara, Silvia Varagnolo, G. Dinesha M. R. Dabera, Kenneth P. Marshall, H. Jessica Pereira, Ross A. Hatton

**Affiliations:** Department of Chemistry, University of Warwick, CV4 7AL Coventry, United Kingdom

## Abstract

We report the findings of a study into the suitability of copper (I) thiocyanate (CuSCN) as a hole-transport layer in inverted photovoltaic (PV) devices based on the black gamma phase (B-γ) of CsSnI_3_ perovskite. Remarkably, when B-γ-CsSnI_3_ perovskite is deposited from a dimethylformamide solution onto a 180–190 nm thick CuSCN film supported on an indium-tin oxide (ITO) electrode, the CuSCN layer is completely displaced leaving a perovskite layer with high uniformity and coverage of the underlying ITO electrode. This finding is confirmed by detailed analysis of the thickness and composition of the film that remains after perovskite deposition, together with photovoltaic device studies. The results of this study show that, whilst CuSCN has proved to be an excellent hole-extraction layer for high performance lead-perovskite and organic photovoltaics, it is unsuitable as a hole-transport layer in inverted B-γ-CsSnI_3_ perovskite photovoltaics processed from solution.

## Introduction

Perovskite photovoltaic (PPV) devices using lead (Pb) halides as the light harvesting semiconductor have shown an unprecedented evolution over the span of less than a decade, with the power conversion efficiency increasing from 3.8% in 2009^1^ to 22.1% in 2016^2^. However, the possibility of lead contamination of the environment due to failure of the device encapsulants or improper disposal at the end of life is a serious concern for commercial exploitation^3–6^. The latter is because lead is a highly toxic element that accumulates in the food chain^7^ and lead perovskites decompose upon exposure to moisture and water to form lead compounds with significant solubility in water^8,9^. Consequently, there is a need for lead-free alternative matched to the needs of photovoltaic (PV) applications. Recent research has shown that tin (Sn) is a potential replacement for Pb in halide perovskites, an element with much lower toxicity than Pb^10^. PPVs based on wholly inorganic tin perovskite PVs are however at a very early stage of development and so to date the highest reported power conversion efficiency is only 6.4%^11^. Much of the dramatic improvement in the power conversion efficiency of lead PPVs has resulted from identification and optimisation of the electron transport layer (ETL) and hole transport layer (HTL)^12^ which interface the light harvesting perovskite layer with the electrodes. These interfacial layers are critically important because they: (i) enable the optimization of the light distribution in the device; (ii) facilitate efficient and selective charge extraction, by minimizing the barrier to the extraction of one carrier type, whilst presenting a large barrier to extraction of the charge carriers with opposite polarity carrier; (iii) physically separate the metal electrode form the perovskite layer, blocking the adverse reactions between them.

In the context of Pb-PPVs the doped organic semiconductor spiro-OMeTAD [2,2′7,7′-tetrakis(N,N-di-p-methoxyphenyl-amine)9,9′-spiro-bifluorene] has proved to be the best HTL to date in terms of the device power conversion efficiency^13^. It is however recognized that the high cost and instability of this material will negatively impact the prospects of commercialization and so there is a need to identify lower cost alternative HTLs. In addition to offering long term stability, the charge transport layer at the transparent electrode must also offer high optical transparency, and so there is interest in using wide band gap wholly inorganic HTLs^14^ including NiO^15^, CuI^16^, Cs_2_SnI_6_^17^ and copper (I) thiocyanate (CuSCN)^18–22^. Among these CuSCN has proved to be a particularly effective HTL for high performance Pb-PPVs with both a conventional^12^ and inverted device architecture^18^, and also for high performance organic PVs^22^. The success of this material as a HTL is due to its wide band gap (≥3.50 eV), excellent solution processability at room temperature and high hole-mobility combined with an ionization potential suitable for the efficient extraction of holes^20,22^. Herein, we report the findings of an investigation into the suitably of CuSCN as a HTL in inverted PV devices based on B-γ-CsSnI_3_, a semiconductor that is attracting considerable attention because it offers near ideal optoelectronic properties for a single junction PV devices and can be processed at room temperature from dimethylformamide (DMF) solutions of CsI and SnI_2_^23^.

**Figure 1:**
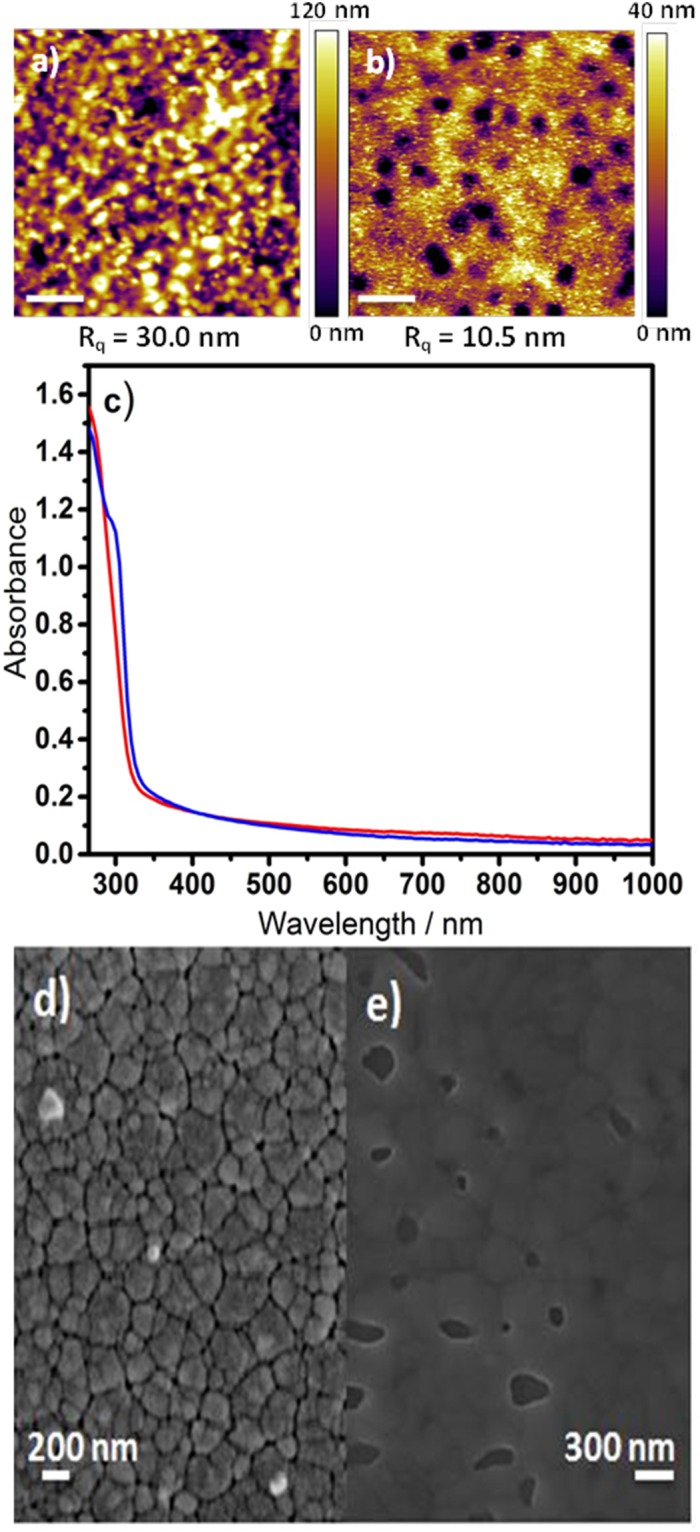
(Top): AFM image of surface topography of CuSCN film before (**a**) and after (**b**) DMF treatment. (**c**) Electronic absorption spectra of a spin cast CuSCN film (180–190 nm) on quartz before (red) and after (blue) washing with DMF by spin casting using the same deposition parameters as used for perovskite film deposition. (Bottom): SEM images of B-γ-CsSnI_3_ + 10 mol% SnCl_2_ deposited onto ITO glass with (**d**) and without (**e**) a CuSCN layer.

In this study diethylsulfide was the solvent of choice for CuSCN deposition because it has been shown to be an excellent solvent for the formation of CuSCN films for electronic applications^18,22^. A solution concentration of 50 mg/ml deposited at room temperature yielded a film thickness of 180–190 nm as measured by step height analysis using atomic force microscopy (AFM): Supporting Information Figure S1. In the first instance these films were used as the HTL in inverted PV devices with the simplified structure: ITO glass| CuSCN| B-γ-CsSnI_3_ + SnCl_2_ (10 mol% excess) |phenyl-C_60_-butyric acid methyl ester (PC_61_BM)|bathocuproine (BCP)|Al. The B-γ-CsSnI_3_ layer was deposited from DMF solutions at room temperature, with 10 mol% excess SnCl_2_ added as a source of excess Sn, according to our previously reported optimized procedure^23^. In a dry nitrogen-filled glovebox CsI, SnI_2_ and SnCl_2_ were mixed together in 1:1:0.1 molar ratio. To this mixture DMF was added to make an 8 wt% solution (total mass of solids), which was stirred overnight before use. To deposit films, two drops of solution were cast onto a substrate spinning at 4,000 rpm for 60 s. To confirm that DMF is an orthogonal solvent for CuSCN a 180–190 nm thick film of CuSCN was washed with DMF using the same spin-coating procedure as used for perovskite film deposition. The AFM images in Fig. 1(a,b) show that this results in significant smoothing of the surface of the CuSCN because the root-mean-square surface roughness (R_q_), measured over an area of 10 μm × 10 μm is reduced from ∼30 nm before spin casting DMF to ∼10 nm after washing with DMF by spin casting. This reduction in surface roughness is consistent with partial dissolution of the CuSCN layer during DMF washing and its possible (partial) removal. However, it is clear from Fig. 1(c) that there is no reduction in the intensity of electronic absorption spectrum, which shows that whilst CuSCN is partially dissolved in DMF it is locally redistributed rather than being washed away. The emergence of the shoulder at a wavelength of ∼300 nm in the absorption spectrum shows that washing with DMF does however significantly improve the crystallinity of the CuSCN film^19^.

**Figure 2:**
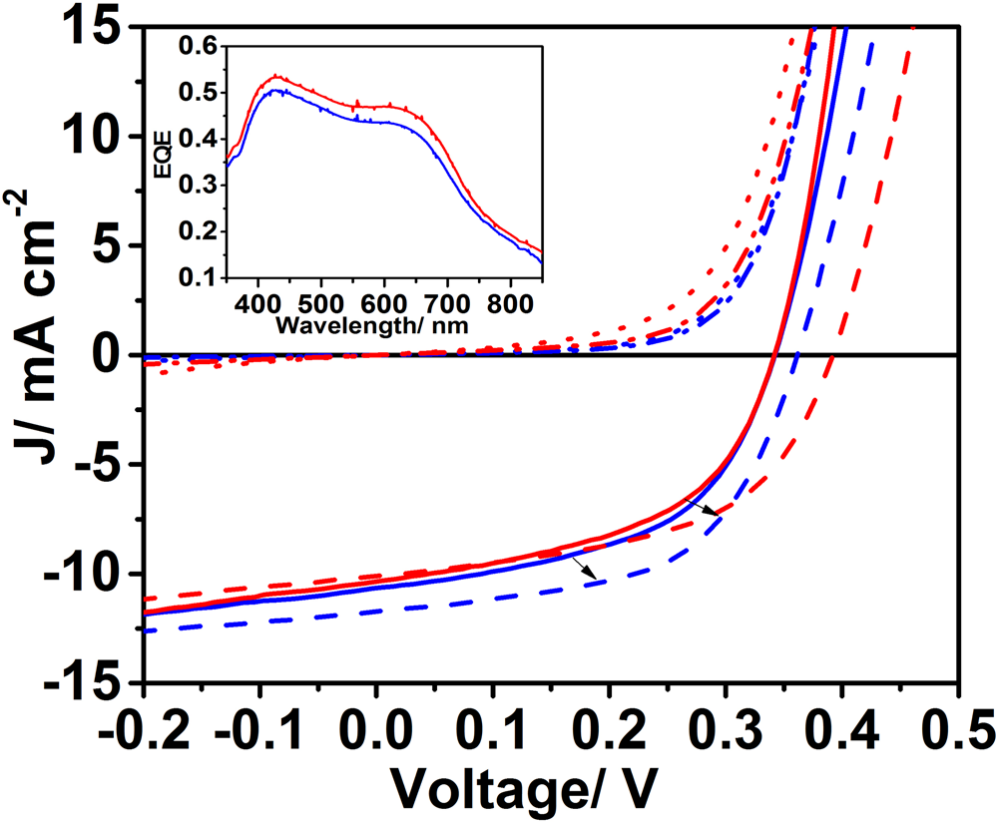
Typical current-voltage (*JV*) characteristics for PPVs with the structure: ITO glass|HTL| B-γ-CsSnI_3_ + 10 mol% SnCl_2_|PC_61_BM|BCP|Al, with (blue) and without (red) a CuSCN HTL layer, immediately after fabrication (solid lines) and after 28 days storage under nitrogen (dashed lines). Inset: Typical external quantum efficiency (EQE) spectra for devices with (blue) and without (red) a CuSCN HTL. All device fabrication and testing was performed in a nitrogen filled glovebox (<1 ppm H_2_O and O_2_).

B-γ-CsSnI_3_ perovskite films deposited from 8 wt% DMF solutions by spin coating directly onto CuSCN supported on ITO glass have a high surface coverage with improved uniformity as compared to perovskite films deposited directly onto ITO glass (Fig. 2(d,e) and Supporting Information Figure S2). Measurements of the perovskite film work function made using a Kelvin probe give the same work function for B-γ-CsSnI_3_ films spun onto freshly cleaned ITO glass and ITO glass coated with 180–190 nm film of CuSCN: 4.57 ± 0.01 eV and 4.56 ± 0.02 eV respectively. The work function of a 180–190 nm thick film of CuSCN on ITO is ∼250 meV larger at 4.82 eV. These measurements show that the work function of the B-γ-CsSnI_3_ layer on CuSCN | ITO is consistent with that of B-γ-CsSnI_3_ only, rather than a complex mixture of B-γ-CsSnI_3_ and CuSCN. That is, the perovskite film appears to have completely covered the underlying substrate without intermixing of the perovskite and CuSCN layers and its thickness is sufficient for the measured work function to be that of B-γ-CsSnI_3_.

It is evident from Fig. 2 that there is no significant difference in the short-circuit current density (*J*_*sc*_), open-circuit voltage (*V*_*oc*_), fill factor (*FF*) or shape of the external quantum efficiency (EQE) spectrum for PV devices with and without a CuSCN HTL. Given that the CuSCN layer increases the total semiconductor thickness in the device by up to a factor of 5, it is surprising that the *J*_*sc*_ remains unchanged (8.1 mA cm^−2^ *vs* 8.2 mA cm^−2^) and the shape of the EQE spectrum remains unchanged, since the light distribution in the device would be significantly changed by the inclusion of such a thick additional layer^24^. It is also unexpected that *V*_*oc*_ for the devices with and without CuSCN; 0.31 V and 0.30 V respectively, is essentially unchanged since the CuSCN layer would be expected to reduce the reverse saturation current by blocking the unwanted extraction of electrons by the ITO electrode, giving rise to an increase in *V*_*oc*_^21,23^.

Additionally, devices with and without a CuSCN layer improve significantly with storage under nitrogen: Fig. 2 (dashed lines) and Table 1 (red). We have previously shown that this improvement in device performance upon storage under nitrogen is typical of B-γ-CsSnI_3_ based PV devices without a HTL^23^, and so it is unexpected that PV devices incorporating a CuSCN HTL should exhibit the same evolution in performance with storage.

**Table 1:** Average current-voltage (*JV*) parameters for PPV devices (±one standard deviation) with the structure: ITO glass|CuSCN| B-γ-CsSnI_3_ + 10 mol% SnCl_2_|PC_61_BM|BCP|Al, immediately after fabrication (black) and after 28 days storage under nitrogen (bold).

Device	*J*_*sc*_/(mA cm^−2^)	*V*_*oc*_/V	*FF*	*η* (%)	Champion *η* (%)
**CuSCN**					
n = 12	8.1 ± 1.1	0.31 ± 0.06	0.45 ± 0.08	1.2 ± 0.5	1.90
**n = 12**	**10.1 ± 1.1**	**0.36 ± 0.01**	**0.54 ± 0.05**	**2.0 ± 0.40**	**2.59**
**Reference**					
n = 8	8.2 ± 1.1	0.30 ± 0.07	0.42 ± 0.09	1.2 ± 0.6	1.78
**n = 7**	**10.1 ± 0.5**	**0.37 ± 0.02**	**0.53 ± 0.09**	**2.2 ± 0.6**	**2.45**

**Figure 3:**
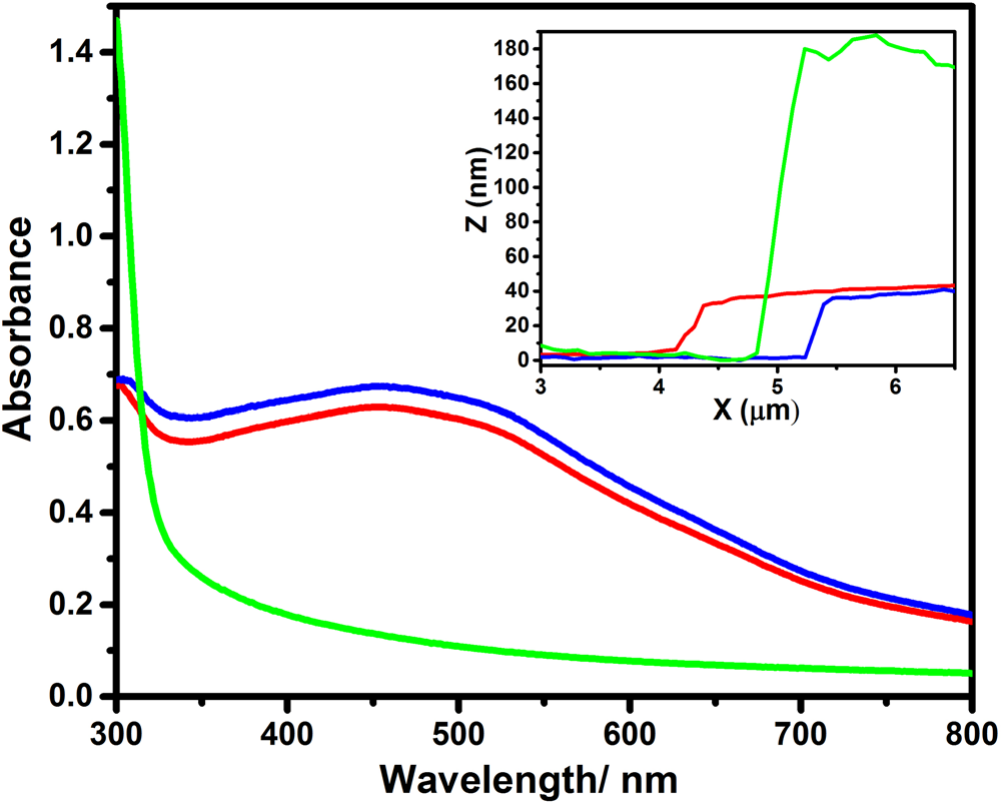
Electronic absorption spectra of a B-γ-CsSnI_3_+ 10 mol% SnCl_2_ film on: glass (red); a bilayer of B-γ-CsSnI_3_+ 10 mol% SnCl_2_|CuSCN on glass (blue); a CuSCN film on glass (green). Inset: Representative AFM image step edge profiles of: B-γ-CsSnI_3_+ 10 mol% SnCl_2_ (red); a bilayer of B-γ-CsSnI_3_+ 10 mol% SnCl_2_|CuSCN (blue); a CuSCN film (green). A statistical analysis of the step edges in scored films is given in Supplementary Information Figure S4.

The simplest explanation for the near identical performance of devices with and without a CuSCN HTL is that there is no change in the energetics at the interface between the perovskite and the ITO electrode and no change in the light distribution within the device, which would require complete displace of the CuSCN film by the perovskite over layer. To investigate this possibility the electronic absorption spectra of the perovskite and the perovskite|CuSCN bilayer was measured: Fig. 3. It is evident from Fig. 3 that the intense absorbance of CuSCN around 300–350 nm is completely lost after perovskite deposition and the spectrum is identical to that of the perovskite on its own, consistent with complete displacement of the CuSCN layer by the perovskite layer. This experiment was also performed without 10  mol% SnCl_2_, and the result was the same (Figure S3) confirming that SnCl_2_ is not responsible for the displacement of the CuSCN layer. To confirm this finding cross-sectional AFM image analysis of scored B-γ-CsSnI_3_+ 10 mol% SnCl_2_ layer and B-γ-CsSnI_3_+ 10% SnCl_2_ | CuSCN bilayer was performed: Fig. 3 (inset) and Supporting Information Figure S4. If the CuSCN and B-γ-CsSnI_3_ thicknesses were additive, the total combined film thickness would be ∼220 nm. However, the measured thickness of the bilayer is the same as that of a B-γ-CsSnI_3_:SnCl_2_ film on its own, consistent with complete displacement of the much thicker underlying CuSCN film.

Corroborating evidence for the complete ejection of the CuSCN layer from the ITO substrate upon perovskite deposition, such that the CuSCN layer is replaced by the perovskite layer is provided by energy dispersive X-ray analysis (EDAX) of the films of CuSCN and B-γ-CsSnI_3_+ 10 mol% SnCl_2_|CuSCN: Figure S5 and Table S1. In both cases EDAX probes the entire film thickness^25^, as is evident from the intense oxygen and indium peaks in both spectra. The intense Cu and S peaks in the spectrum of CuSCN are completely absent in the bi-layer film, consistent with displacement of the CuSCN film. Further corroborating evidence is provided by X-ray photoelectron spectroscopy (XPS) analysis (Figure S6) which probes the top 8–10 nm^23^ of the perovskite film and any of the underlying substrate exposed at the site of grain boundaries or pinholes. Whilst it is evident from the SEM image in Fig. 2(d) that the perovskite film is compact, there is also a high density of grain boundaries. The indium 3d peaks are clearly resolved in the high resolution (HR) XPS spectrum of the perovskite film deposited onto CuSCN | ITO, with a binding energy consistent with that of ITO^26,27^ (Figure S7) confirming that the underlying ITO is probed using this technique. The complete absence of Cu, N and S in the HR-XPS spectra (Figure S6) is consistent with the complete displacement of the CuSCN upon spin casting the perovskite layer. Additionally, X-ray diffraction (XRD) analysis confirms this conclusion, since there is no overlap between the powder patterns of the bilayer and CuSCN (Figure S8) and all of the reflections in the XRD pattern of the bilayer can be assigned to the simulated pattern of B-γ-CsSnI_3_^28^.

To test the generality of this finding we have also considered the case of using dimethyl sulfoxide (DMSO) as the solvent for perovskite film deposition. Notably, unlike for DMF, B-γ-CsSnI_3_ perovskite does not form directly from DMSO solutions at room temperature, but there is rapid crystallization to form a solvated intermediate^29^ which must be annealed for conversion to the perovskite. Figure S9 shows the electronic absorption spectra of the perovskite and the perovskite|CuSCN bilayer, from which it is evident that the intense absorbance of CuSCN around 300–350 nm is completely lost after perovskite deposition, and the spectrum is essentially identical to that of the perovskite only. This conclusion is supported by EDAX analysis of CuSCN film before and after deposition of the perovskite onto the CuSCN film: In the EDAX spectrum of the bilayer (Figure S10) the Cu and S peaks are absent, or at the resolution limit. Corroborating evidence is provided by the XPS analysis of the bilayer deposited from DMSO given in (Figure S11), which shows that Cu, S and N are not present in the film. The presence of intense In 3d peaks with binding energies consistent with ITO^30^ environment (Figure S12) shows that the underlying substrate is visible to this this technique, consistent with the large gaps between crystallites seen in the SEM image of the film (Figure S13). Taken together the experimental evidence therefore shows that the 180–190 nm CuSCN layer is completely by the perovskite film during the deposition process, just as when DMF is used as the solvent.

The complete displacement of the 180–190 nm thick CuSCN film by the perovskite film (which in this case is also only one quarter of the thickness of the CuSCN film) is remarkable, not least because of the high speed of film formation during spin-casting. This finding is attributed to a combination of the increased solvation power of DMF and DMSO for CuSCN, due to the high ionic strength associated with halide and metal ions, together with the tendency for very rapid crystallization either directly to the B-γ-CsSnI_3_ perovskite or a solvated intermediate^29^. Tin perovskites are known to crystallize much more rapidly than their lead analogues due to the high Lewis acidity of Sn^2+ ^^30^, and the enthalpy of formation of the B-γ-CsSnI_3_ perovskite is very low as compared to that of CuSCN (∼0.06 eV^31^ *vs* 1.14 eV^32^), so the process of complete ejection of the CuSCN under layer is evidently a strongly kinetically driven process.

In conclusion, whilst CsSCN has proved to be a low cost HTL for Pb-PPV and high performance organic PVs, here we have shown that is unsuitable as a HTL in solution processed B-γ-CsSnI_3_ based PPV with an inverted device architecture. Unencapsulated PPV based on B-γ-CsSnI_3_ with an inverted architecture have been shown to exhibit promising device stability^23^, although the power conversion efficiency has remained stubbornly low primarily due to a low *V*_*oc*_. The ionization potential of B-γ-CsSnI_3_ is particularly small amongst tin and lead halide perovskites^33^ and so in order to achieve a significantly increased *V*_*oc*_, energetic losses that occur during hole-extraction must be minimized. The identification of a suitable HTL layer, which has hitherto proved elusive^31^, is therefore essential to improve the efficiency of B-γ-CsSnI_3_ based PPVs, and so represents a fertile area of research.

## Methods

### CuSCN film deposition

A solution concentration of 50 mg/ml CuSCN in diethyl sulfide was drop cast onto a freshly cleaned ITO glass substrate spinning at 3000 rpm for 60 seconds.

### B-γ-CsSnI_3_ film deposition

In a dry nitrogen-filled glovebox CsI, SnI_2_ and tin(II) halide were mixed together in 1:1:0.1 molar ratio. To this mixture N,N-dimethylformamide (DMF) was added to make an 8 wt% solution (total mass of solids), which was stirred overnight before use. To deposit films, two drops of solution were cast onto the substrate (sufficient to cover the whole substrate), followed by spinning at 4,000 r.p.m. for 60 s. The B-γ-CsSnI_3_ phase forms immediately upon solvent evaporation.

### Device fabrication

Indium tin oxide (ITO) coated glass slides (Thin Films Devices Inc. 15 ± 3 Ω sq^−1^.) were held in vertical slide holders and ultrasonically agitated in an acetone bath, followed by a high purity water bath with a few drops of surfactant, followed by high purity deionized water only bath, acetone and finally an isopropanol bath. Slides were then UV/O_3_ treated for 15 minutes. Immediately after UV/O_3_ treatment the slides were transferred into a dry nitrogen filled glovebox for CuSCN film deposition followed by deposition of the perovskite and finally the PC_61_BM layer from 13 mg ml^−1^ chlorobenzene solution using a spin speed of 1500 rpm. This was followed by thermal evaporation of 6 nm bathocuproine (BCP) deposited at 0.5 Å s^−1^ and then 60 nm of Al deposited at 1 Å s^−1^. Thermal evaporation was performed at a pressure of 1 × 10^−5^ mbar with substrate rotation. The Al electrode was deposited through a shadow mask to make six devices per slide, each with an area of 6 mm^2^.

### PV device testing

Device testing was performed in the same glove box as used for device fabrication using a solar simulator inside the glove box. Current density–voltage (*J*–*V*) curves were measured using a Keithley 2400 source-meter under AM1.5 G solar illumination at 100 mW cm^2^ (1 sun), scanned from 1 V to + 1 V at 0.1 Vs^-1^. External quantum efficiency (EQE) measurements were carried out using a Sciencetech SF150 xenon arc lamp and a PTI monochromator, with the monochromatic light intensity calibrated using a Si photodiode (Newport 818-UV). The incoming monochromatic light was chopped at 180 Hz. For signal measurement a Stanford Research Systems SR 830 lock-in amplifier was used. *J*–*V* and EQE measurements were made using custom LabVIEW programs.

### Electronic absorption spectroscopy

Ultraviolet/visible/near-infrared spectra were measured for optically thin films of CuSCN, B-γ-CsSnI_3_ and bilayer of CuSCN B-γ-CsSnI_3_ on glass or quartz substrates.

### Atomic force microscopy (AFM)

AFM imaging was performed in tapping mode using an Asylum Research MFP – 3D to determine the step height of the films and morphologies.

### Scanning electron microscopy (SEM)

SEM imaging was performed using a Zeiss SUPRA 55VP field emission gun SEM.

### X-ray photoelectron spectroscopy (XPS)

XPS analysis was performed using a Kratos AXIS Ultra DLD. Samples were unavoidably exposed to air for approximately 1 min during transfer from an air-tight box to the vacuum chamber of the instrument. XPS measurements were carried out in an ultrahigh vacuum system with a base pressure of 5 × 10^−11^ mbar. The sample was excited with X-rays from a monochromated Al Kα source (hν = 1,486.7 eV), with the photoelectrons being detected at a 90° take-off angle. The sputtering was carried out at room temperature using a Minibeam I ion gun (Kratos Analytical). A beam of 4 keV Ar^+^ ions was incidenton a 3 × 3 mm area of the sample surface. Curve fitting was performed using the CasaXPS package, incorporating Voigt (mixed Gaussian Lorentzian) line shapes and a Shirley background.

### X-ray diffraction (XRD)

XRD was performed on thin films of CuSCN prepared from diethyl sulphide solution and a bilayer of CuSCN and B-γ-CsSnI_3_ prepared from 8 wt% (total solids) DMF solution deposited onto a glass substrate (13 × 13 mm^2^) spinning at 4000 rpm for 60 seconds. Measurements were made on a Panalytical X’Pert Pro MRD equipped with an Anton Paar DHS 1100 domed stage under a flow of N_2_. Simulated diffraction patterns were calculated using the program Mercury 3.122 using CIFs from the Inorganic Crystal Structure Database (ICSD).

## Electronic supplementary material

Supporting Information

## Data Availability

All data supporting this study are provided as supplementary information accompanying this paper.
