# Supplementary material for: Assessing the suitability of copper thiocyanate as a hole-transport layer in inverted CsSnI3 perovskite photovoltaics
Source: Sci Rep. 2018 Oct 24;8:15722. doi: 10.1038/s41598-018-33987-7 (PMC6200744; doi:10.1038/s41598-018-33987-7)
Supplement: Supplementary file 1 — Supporting Information [file 41598_2018_33987_MOESM1_ESM.docx]

**Assessing the suitability of copper thiocyanate as a hole-transport layer in inverted CsSnI_3_ perovskite photovoltaics**

Anjana Wijesekara^1^, Silvia Varagnolo^1^, G Dinesha M R Dabera^1^, Kenneth P. Marshall^1^, H. Jessica Pereira^1^ and Ross A. Hatton^1^*

[1] Department of Chemistry, University of Warwick, CV4 7AL, Coventry, United Kingdom

*Corresponding author: Ross.Hatton@warwick.ac.uk

**Supporting Information**

**(d)**


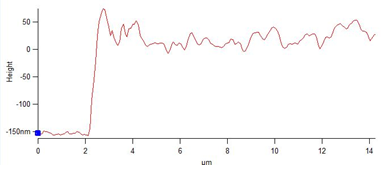

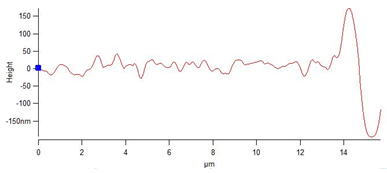

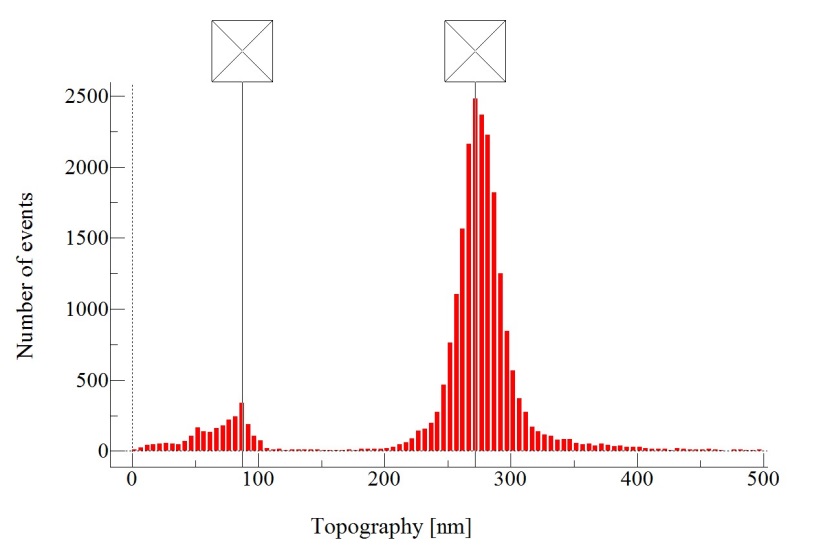

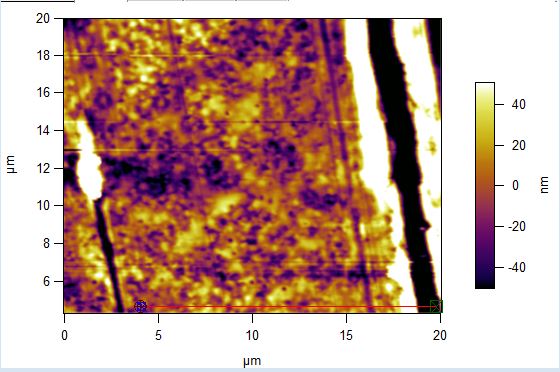


**(a)**

**(b)**

**(c)**


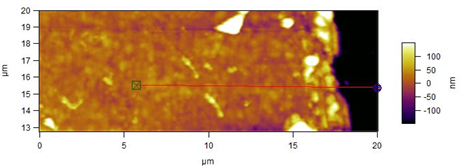


**(e)**

**(d)**

185.4 nm

**Figure S1|**: (a)-(e) AFM step height analysis for scored CuSCN films spin cast from 50 mg / ml diethylsulfide solutions onto ITO glass.

a)


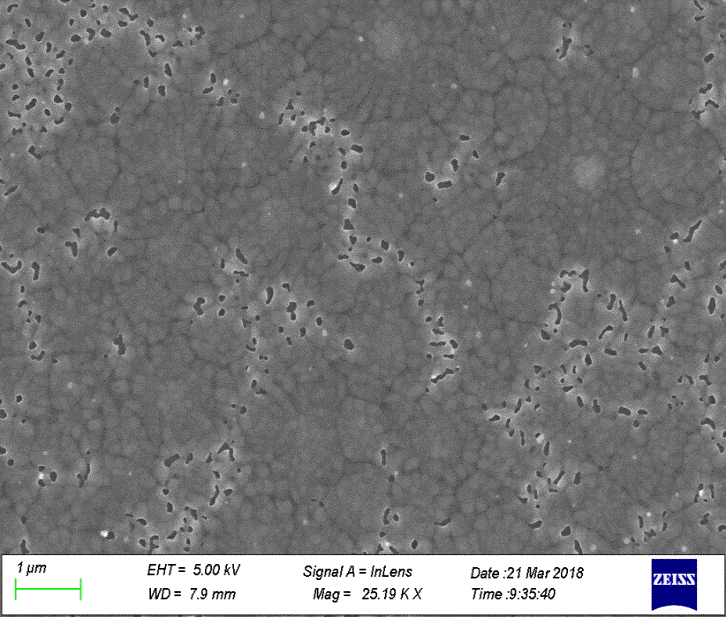

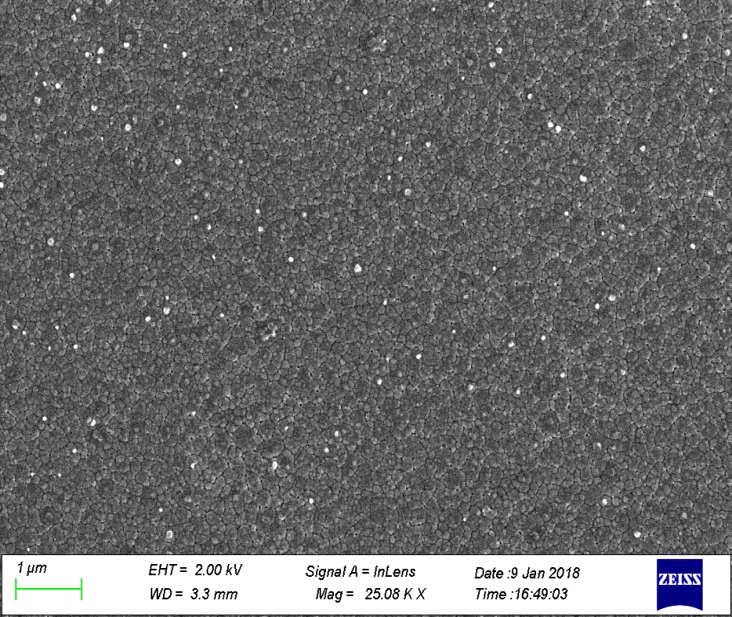


**1 μm**

**(b)**

**(a)**

**Figure S2**| SEM images of **(a)** CsSnI_3_ + 10% SnCl_2_ spin cast onto CuSCN coated ITO glass and **(b)** CsSnI_3_ + 10% SnCl_2_ deposited onto ITO glass without a CuSCN layer.


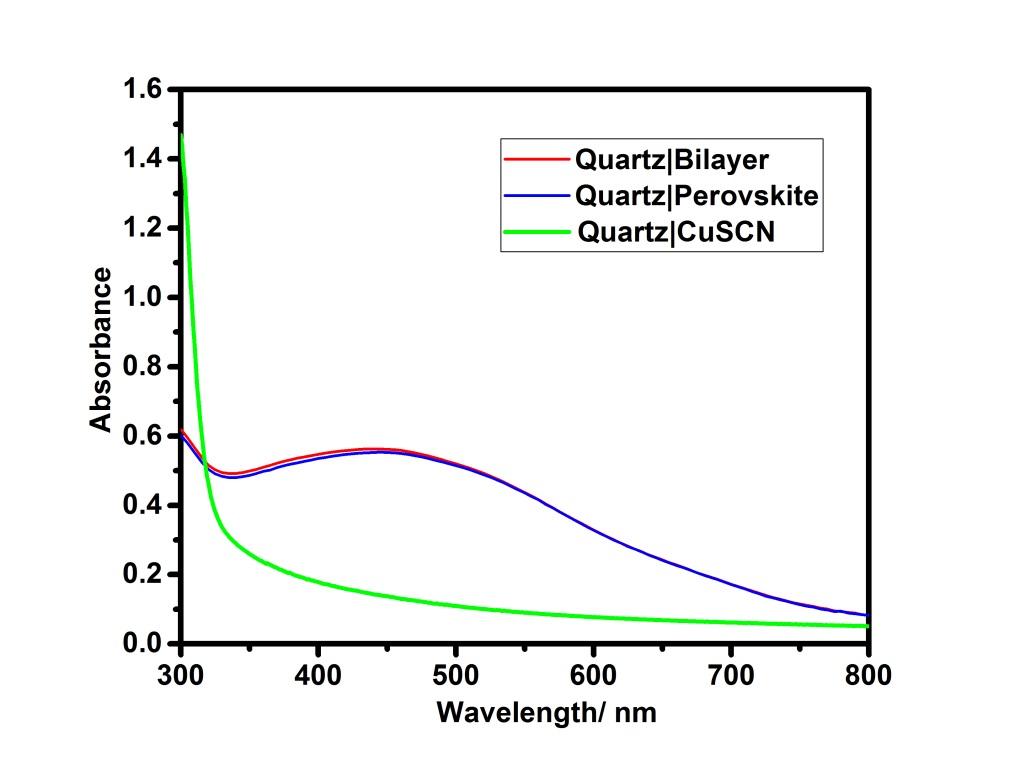


**Figure S3**| Electronic absorption spectra of a CsSnI_3_ film (blue); a bilayer of CsSnI_3_|CuSCN on quartz (red); a CuSCN film (green). All films are supported on quartz substrates.


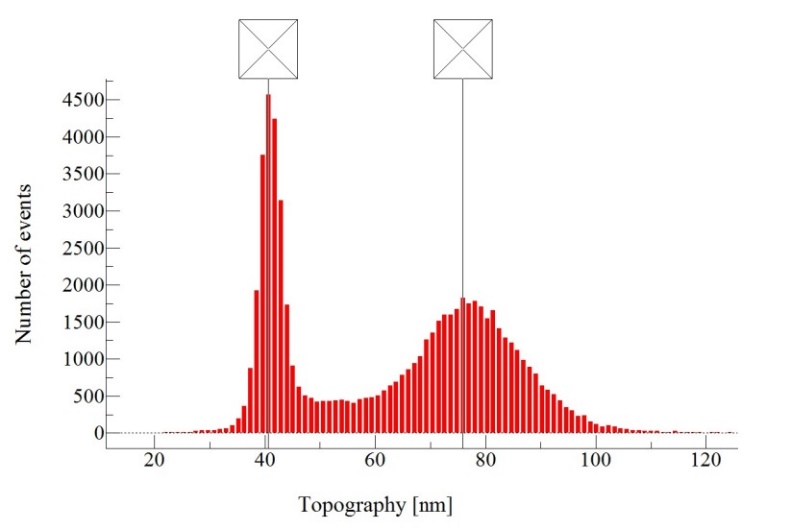

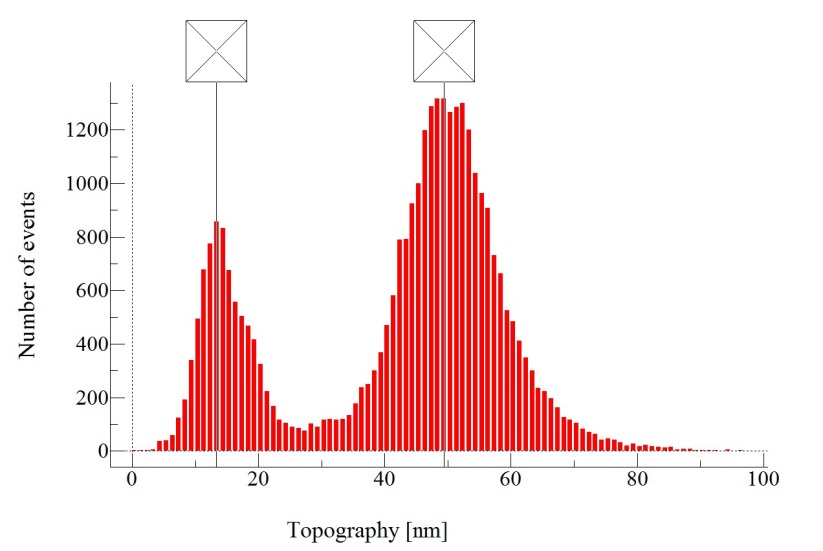


**(a)**

**(b)**

**Figure S4**| Step edge analysis of scored films of: ITO|CuSCN| CsSnI_3_:SnCl_2_ **(a)**; and ITO| CsSnI_3_:SnCl_2_ **(b)**.

Thickness = 35.28 nm

Thickness = 35.28 nm

**Table S1**: Atomic composition determined from EDAX analysis of a film of CsSnI_3_:SnCl_2_ deposited directly onto a ∼190 nm thick CuSCN film and ∼190 nm thick CuSCN film supported on an ITO glass substrate.

| Composition (%) | Cs | Sn | I | Cu | S | In | N |
| --- | --- | --- | --- | --- | --- | --- | --- |
| CuSCN\|ITO | 0.00 | 0.00 | 0.00 | 16.45 | 12.60 | 14.20 | 16.92 |
| CsSnI_3_\|CuSCN\|ITO | 3.80 | 7.93 | 9.45 | 0.00 | 0.00 | 46.66 | 2.10 |


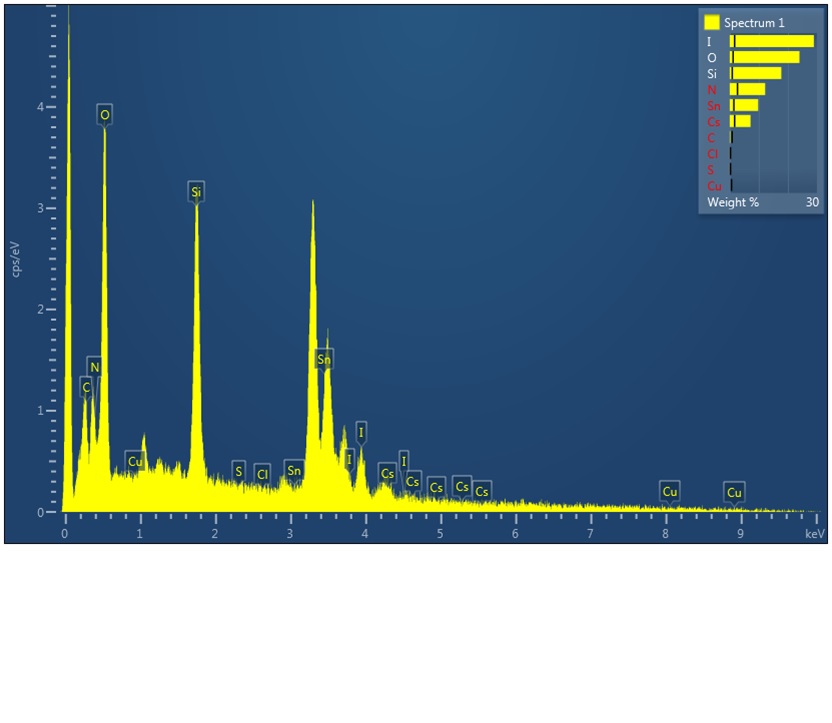


**(a)**


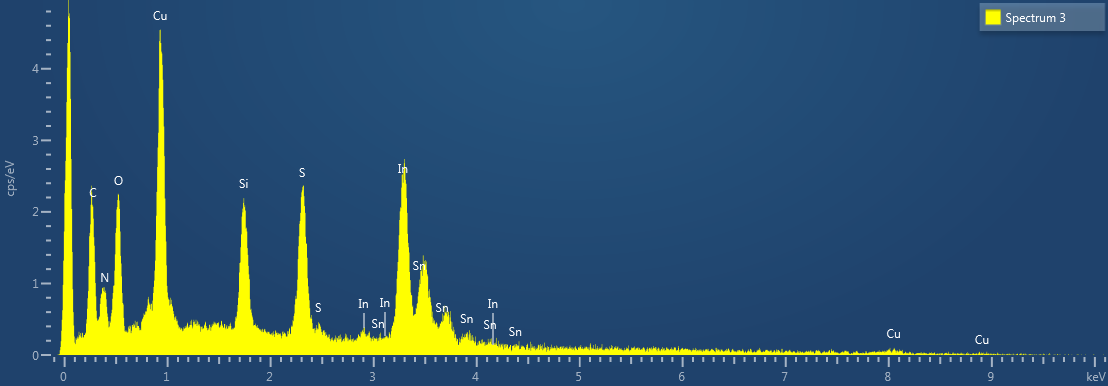


**(b)**

**Figure S5:** Energy-dispersive X-ray spectrum of a film of: **(a)** CsSnI_3_:SnCl_2_ deposited onto a ∼190 nm thick CuSCN film supported on ITO glass; **(b)** ∼190 nm thick CuSCN film. In (a) the peak corresponding to indium is at 3.28 keV


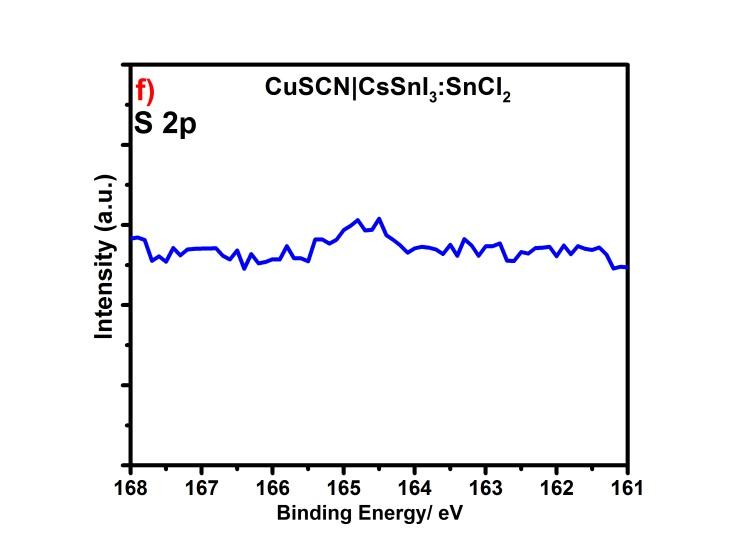

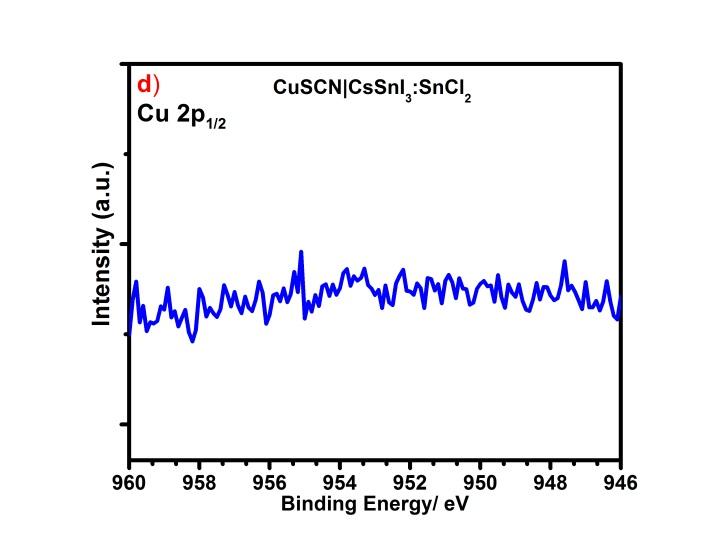

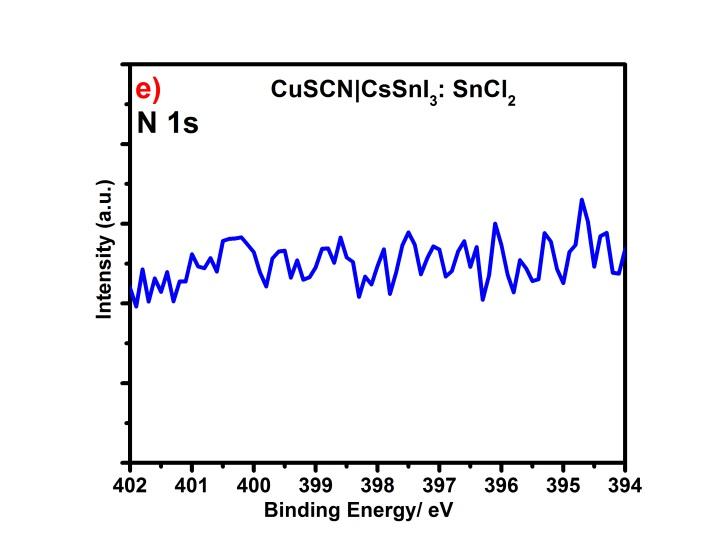

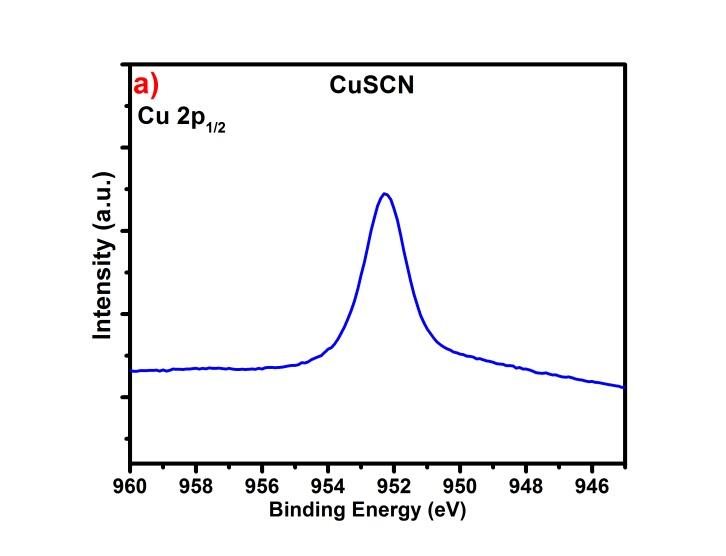

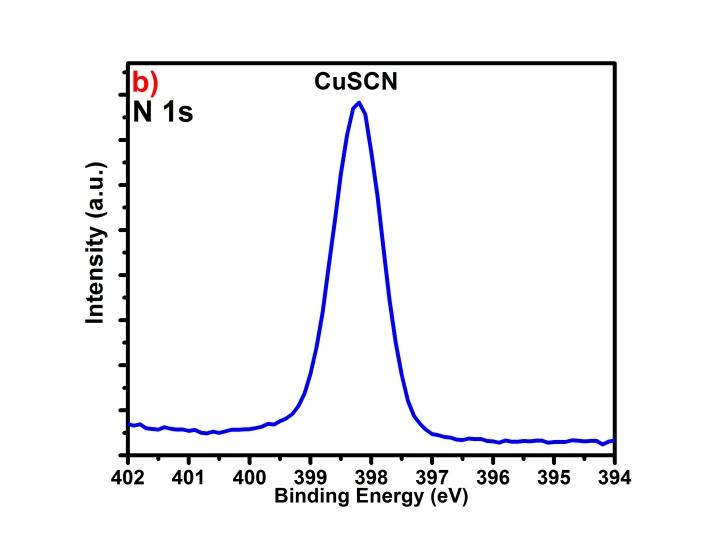

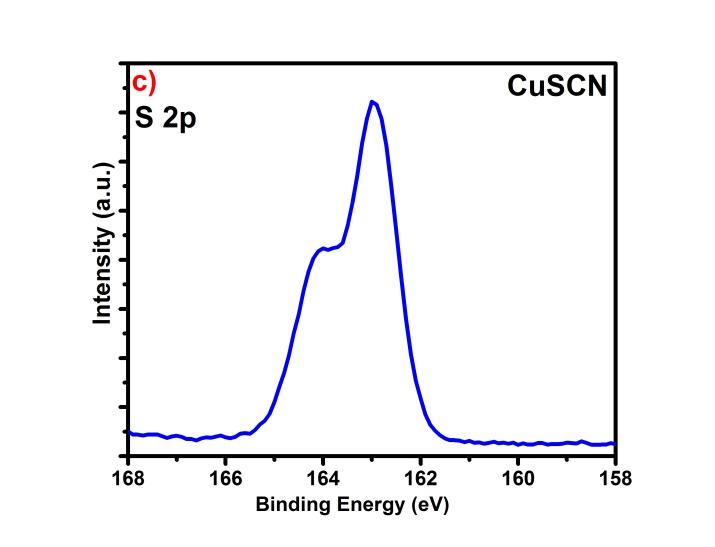


**(b)**


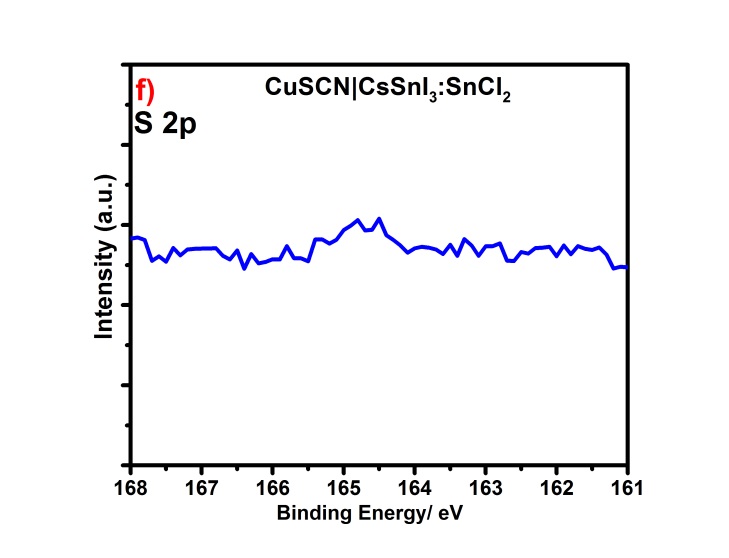

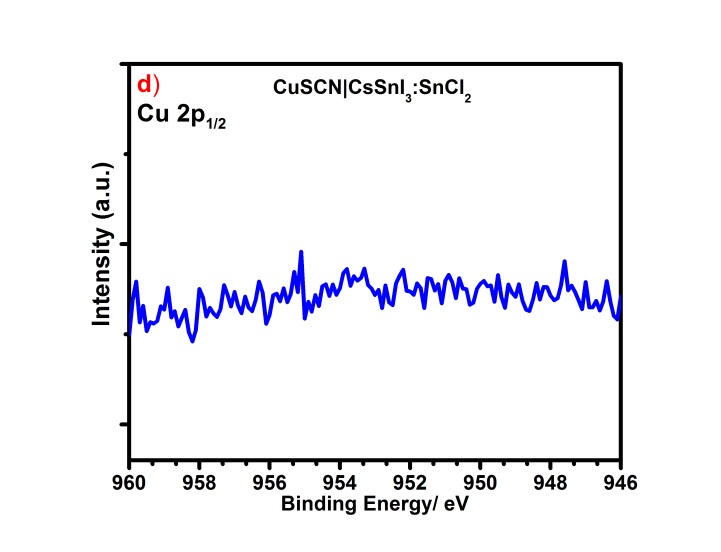

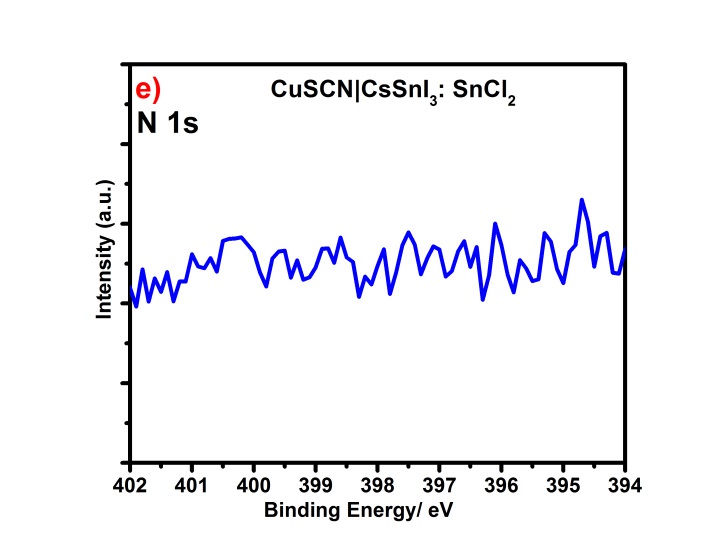

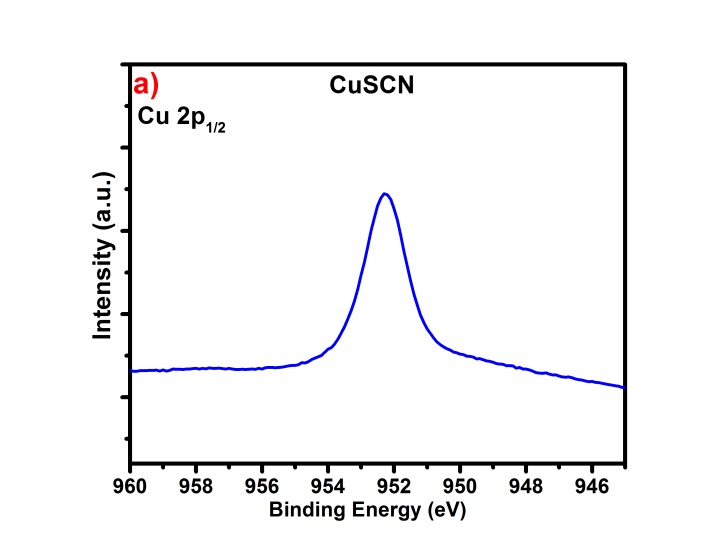

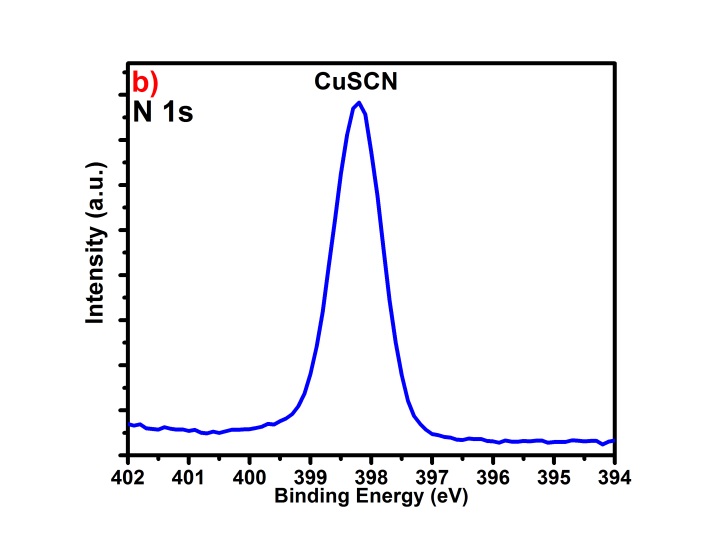

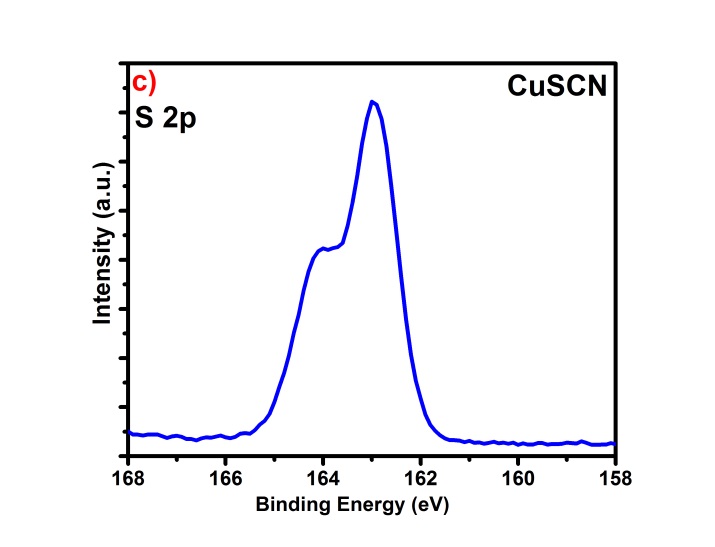


**Figure S6|** High resolution (HR) XPS spectra of the: **(a)** Cu 2p_1/2_ region for a film of CuSCN. The Cu 2p_3/2_ peak is not shown because it overlaps with the I 3p peak.; **(b)** N 1s region for a thin film of CuSCN.; **(c)** S 2p region for a film of CuSCN.; **(d)** Cu 2p_1/2_ region for a film of CsSnI_3_:SnCl_2_ deposited onto CuSCN.; **(e)** N 1s region for a film of CsSnI_3_:SnCl_2_ deposited onto CuSCN.; **(f)** S 2p region for a film of CsSnI_3_:SnCl_2_ deposited onto CuSCN.


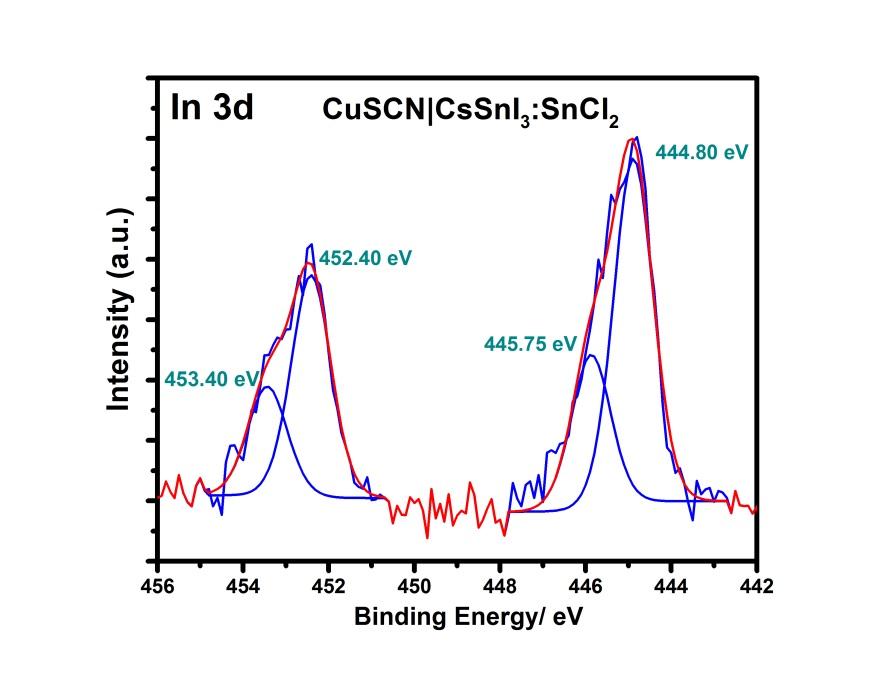


**Figure S7|** Peak-fitted HRXPS spectra of the In 3d region for a film of CsSnI_3_:SnCl_2_ deposited onto CuSCN | ITO glass.


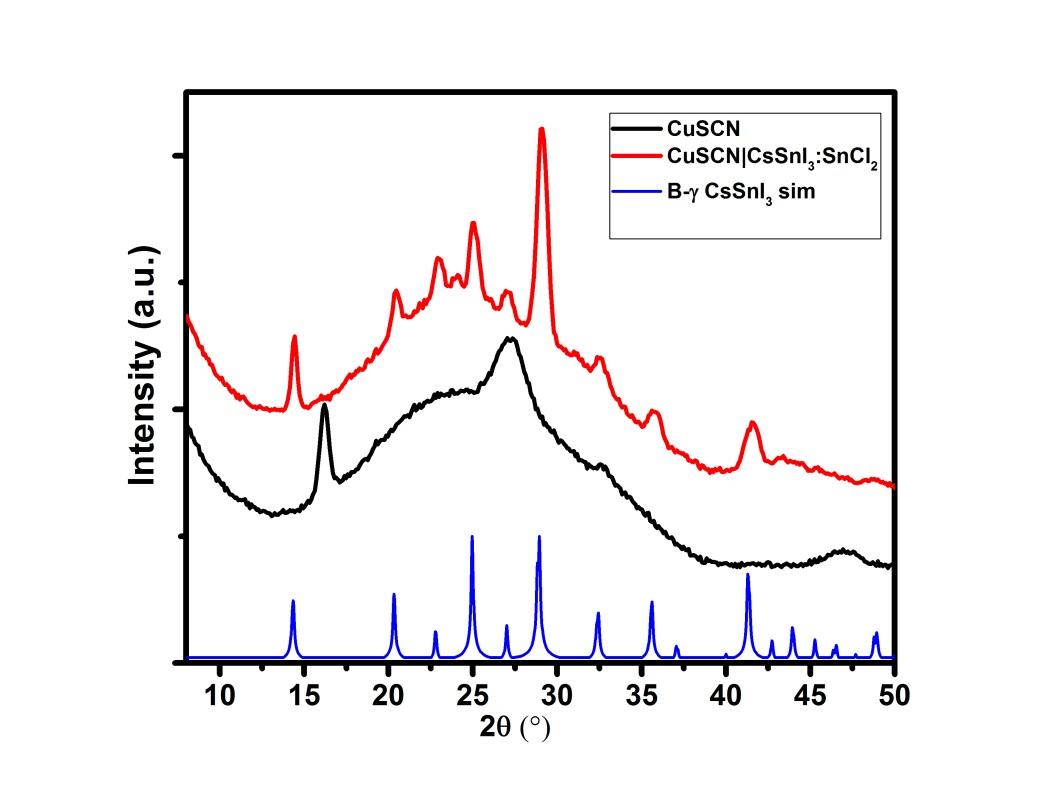


**Figure S8|** XRD pattern of CsSnI_3_:SnCl_2_ on a CuSCN | glass substrate, and a film of CuSCN on glass. Simulated patterns of CsSnI_3_ (calculated using 262925-ICSD). Spectra have been offset vertically along the y-axis for clarity.


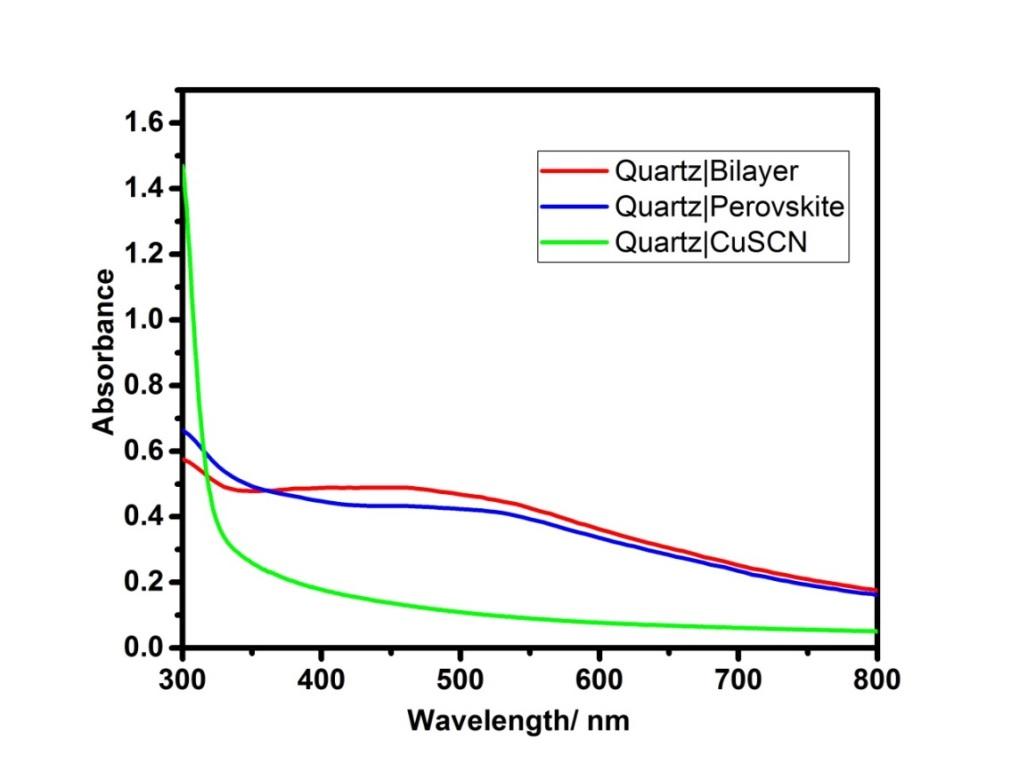


**Figure S9**| Electronic absorption spectra of a film of CsSnI_3_:SnCl_2_ deposited from a DMSO solution (blue); a bilayer of CsSnI_3_:SnCl_2_|CuSCN (red); a CuSCN film (green). All films are supported on quartz substrates.


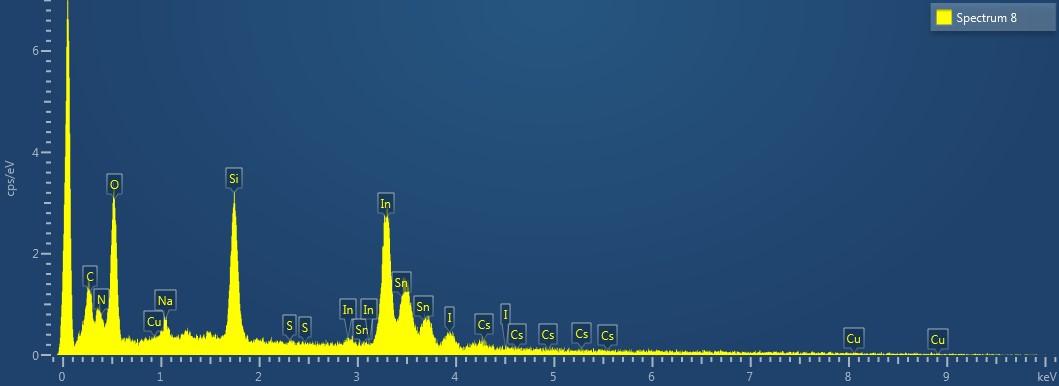


**Figure S10|** Energy-dispersive X-ray spectrum of a film of a CsSnI_3_:SnCl_2_ deposited from DMSO onto a ∼190 nm thick CuSCN film supported on ITO glass substrate.

**Figure S11|** XPS spectra of the: Cu 2p_1/2_ (a), N 1s (b) and S 2p (c) regions for a film of CsSnI_3_:SnCl_2_ deposited from DMSO onto CuSCN/ITO glass.


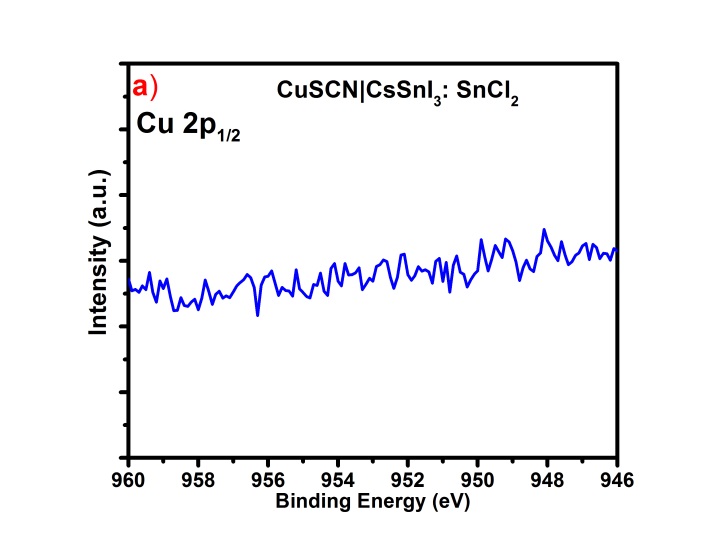

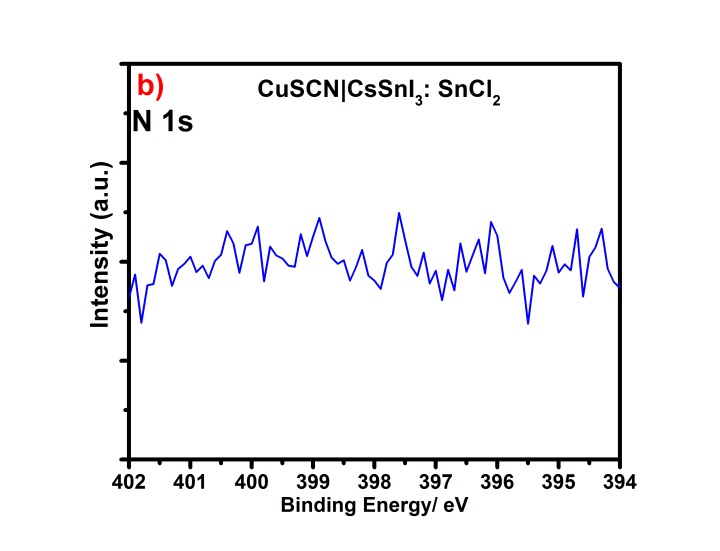

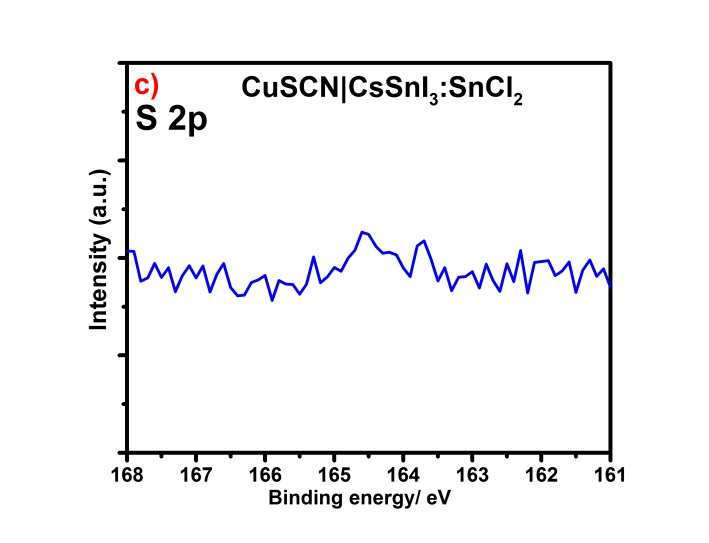


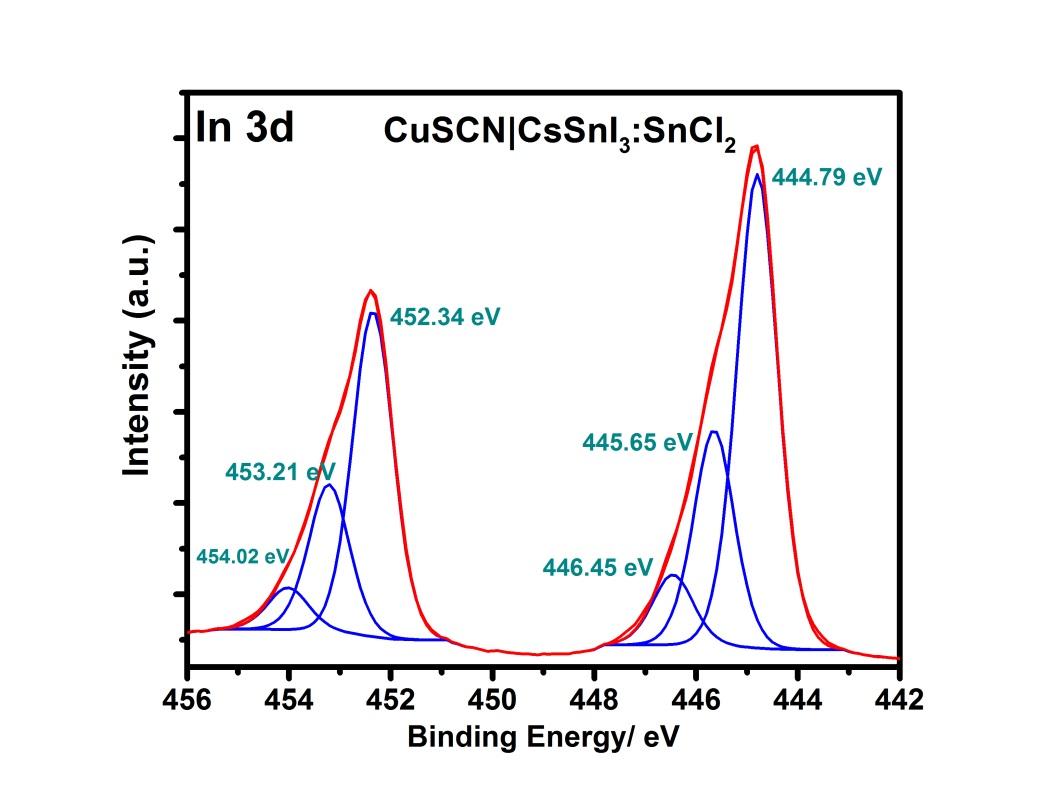


**Figure S12|** High resolution XPS spectra of an In 3d environment for a film of CsSnI_3_:SnCl_2_ deposited from DMSO onto CuSCN supported on ITO glass.


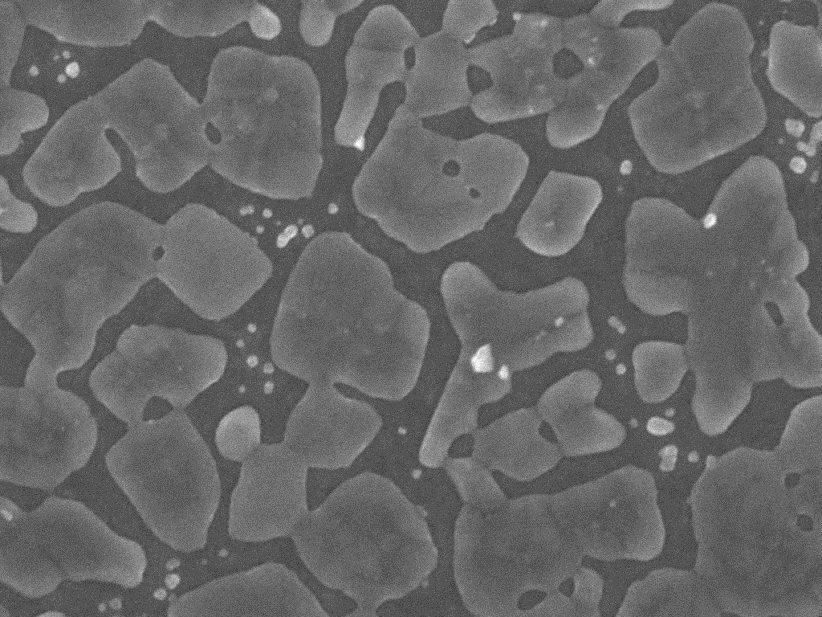


**200 nm**

**Figure S13**| SEM images of CsSnI_3_ + 10% SnCl_2_ spin cast from DMSO onto CuSCN coated ITO glass.
